# Better Prognosis of Patients with Glioma Expressing FGF2-Dependent PDGFRA Irrespective of Morphological Diagnosis

**DOI:** 10.1371/journal.pone.0061556

**Published:** 2013-04-22

**Authors:** Dongfeng Chen, Annette Persson, Yingyu Sun, Leif G. Salford, David Gisselsson Nord, Elisabet Englund, Tao Jiang, Xiaolong Fan

**Affiliations:** 1 The Rausing Laboratory, Department of Neurosurgery, Lund University, Lund, Sweden; 2 Department of Pathology, Lund University, Lund, Sweden; 3 Department of Clinical Genetics, Lund University, Lund, Sweden; 4 Glioma Center, Department of Neurosurgery, Beijing Tiantan Hospital, Capital Medical University, Beijing, China; 5 Beijing Key Laboratory of Gene Resource and Molecular Development, Laboratory of Neuroscience and Brain Development, Beijing Normal University, Beijing, China; University of Pécs Medical School, Hungary

## Abstract

Signaling of platelet derived growth factor receptor alpha (PDGFRA) is critically involved in the development of gliomas. However, the clinical relevance of PDGFRA expression in glioma subtypes and the mechanisms of PDGFRA expression in gliomas have been controversial. Under the supervision of morphological diagnosis, analysis of the GSE16011 and the Repository of Molecular Brain Neoplasia Data (Rembrandt) set revealed enriched PDGFRA expression in low-grade gliomas. However, gliomas with the top 25% of PDGFRA expression levels contained nearly all morphological subtypes, which was associated with frequent *IDH1* mutation, 1p LOH, 19q LOH, less EGFR amplification, younger age at disease onset and better survival compared to those gliomas with lower levels of PDGFRA expression. SNP analysis in Rembrandt data set and FISH analysis in eleven low passage glioma cell lines showed infrequent amplification of *PDGFRA*. Using *in vitro* culture of these low passage glioma cells, we tested the hypothesis of gliogenic factor dependent expression of PDGFRA in glioma cells. Fibroblast growth factor 2 (FGF2) was able to maintain PDGFRA expression in glioma cells. FGF2 also induced PDGFRA expression in glioma cells with low or non-detectable PDGFRA expression. FGF2-dependent maintenance of PDGFRA expression was concordant with the maintenance of a subset of gliogenic genes and higher rates of cell proliferation. Further, concordant expression patterns of FGF2 and PDGFRA were detected in glioma samples by immunohistochemical staining. Our findings suggest a role of FGF2 in regulating PDGFRA expression in the subset of gliomas with younger age at disease onset and longer patient survival regardless of their morphological diagnosis.

## Introduction

Gliomas are the most common primary tumors in adult central nervous system. According to the World Health Organization classification scheme, gliomas are morphologically diagnosed as astrocytic, oligodendroglial, and mixed oligoastrocytic tumors, which are further subdivided into I to IV malignant grades based on the extent of cell proliferation, angiogenesis and necrosis [1]. Despite intensive efforts in mechanistic and clinical investigations, the prognosis of glioma patients remains poor. Patients with glioblastoma multiforme (GBM) have a survival time of only up to 2 years after diagnosis [2]. Low-grade gliomas will eventually progress into high-grade gliomas, although with varying kinetics among the patients. A large number of studies have indicated a resemblance between glioma cells and immature neural cells, including neural stem cells and glial progenitor cells [3], [4], [5]. Understanding the mechanisms that are responsible for these resemblances may clarify causes for the abnormal proliferation, migration and differentiation features of glioma cells, and thereby identify new therapeutic targets.

Glioma cells are maintained by interplay between the intrinsic gene expression profiles of glioma cells and the niche environment. Gliogenic growth factors and their receptors are frequently over-expressed in gliomas [6], [7], [8]. PDGFRA is a characteristic marker of oligodendrocyte progenitor cells (OPCs) [9], [10], [11], [12] and its disruption results in diminished oligodendrogenesis [13]. Previous studies have also demonstrated crucial roles of PDGFRA signaling in gliomagenesis and shown that it is overexpressed in 30% of human gliomas [5], [7], [14], [15]. In particular, autocrine stimulation of PDGFRA signaling is suggested to be important for glioma initiation and progression [15], [16]. In mice, PDGFB overexpression in neonatal CNS or adult CNS, either by the transgenic approach on a p53^−/−^ background [17], or by the retroviral gene transfer approach [16], [18], [19], generated glioma-like tumor growth. Interestingly, tumors generated in these models all expressed PDGFRA [17], [18], [20]. Inhibition of PDGFRA signaling resulted in a reversion of transformed phenotype in glioma cell lines [21], or a reversion from high-grade to lower grade tumor histology in mouse model [22]. However, compared to the established close association between EGFR high expression and *EGFR* gene amplification and mutation [23], the regulation mechanism for PDGFRA expression in glioma cells is yet unclear [5], [24]. Further, the occurrence and clinical relevance of PDGFRA expression in different glioma subtypes is debated and controversial as both enhanced as well as negative expression patterns have been reported in GBM [8], [14], [24]. Recently, enriched PDGFRA expression was reported in the proneural GBM subtype which was seen to be associated with a better prognosis compared to the other molecular subtypes of GBM [25], [26], while other studies reported no correlation between PDGFRA expression and clinical-pathological parameters of glioma patients [14].

In this report, we hypothesize that PDGFRA expression is regulated by gliogenic factors in gliomas. Using large glioma gene expression databases, we found that the expression of both PDGFRA and FGF2 were enriched in low-grade gliomas. Using *in vitro* cell culture combined with microarray gene expression analysis, we demonstrated that FGF2 dependent glioma cell growth was concomitant with maintained expression of a subset of gliogenic genes, including *PDGFRA*. Clinically, enriched PDGFRA expression in low- as well as in high-grade glioma samples was associated with younger age at disease onset and better survival outcome of patients. Our findings indicated FGF2 signaling as a potential intervening target in glioma subset with enriched PDGFRA expression.

## Materials and Methods

### Ethics Statement

The collection and use of the human tissues in this study were performed after obtaining written consent from all participants, in accordance with a study protocol approved by The Regional Ethical Review Board in Lund, Sweden with the permission H15 642/2008. Glioma biopsies of fresh tumor tissue were obtained from patients operated at the Clinic of Neurosurgery, Lund University Hospital, Sweden. Glioma tissue blocks derived from surgical excision biopsies were obtained from the archive of Department of Pathology, Lund University Hospital.

### Microarray data and analysis of microarray gene expression data

Total cellular RNA was extracted and purified using the RNeasy Mini kit (Qiagen) according to the manufacturer's instructions. The quality of total RNA and integrity of rRNA were determined using Agilent 2100 Bioanalyzer (Agilent Technologies). Cells from two GBM cell lines (L1 and L2) cultured under conditions with FGF2 (+FGF2) or without FGF2 (−FGF2) support were used. Cells were harvested when PDGFRA expression in −FGF2 condition was undetectable by flow cytometric analysis. Each condition contained three biological replicates. The hybridizations were performed at SCIBLU of Lund University using Affymetrix arrays (GeneChip® Human Gene ST Arrays, Affymetrix).

Microarray data from GSE16011 (containing 244 gliomas of all major morphological subtypes and 8 controls), GSE4290 (containing 157 gliomas of all major morphological subtypes and 23 controls) and the Rembrandt (containing 404 gliomas of all major known morphological subtypes and 21 controls), were gathered from published studies [27], [28], [29]. The CEL files for GSE16011 and the Rembrandt data set were separately merged and computed with RMAExpress tool. The expression data were normalized according to quantile normalization, and expressed in natural scale. FGF2 or PDGFRA expression was analyzed using SPSS software in GSE16011 and Rembrandt data sets, respectively. Qlucore Omics Explore 2.2 (Qlucore AB, Lund, Sweden) was used for gene expression clustering analysis.

A gliogenesis gene list of 172 genes was first compiled from gene ontology GO:0042063 and transcriptional control of oligodendrogenesis [30]. Subsequently, differential expression of gliogenesis genes in glioma samples was evaluated in GSE4290 [29], which includes 157 gliomas of all major morphological subtypes and malignant grades and 23 epileptic brain samples as control. Seventy-eight gliogenesis genes differentially enriched in glioma samples (compared to non-tumor brain samples) in GSE4290 were selected as glioma-relevant gliogenesis genes (**Figure S1**).

### SNP-array analysis

Copy number analysis was performed on 205 gliomas from the Rembrandt data set [28]. The raw *HindIII* CEL files from the 50K single nucleotide polymorphism (SNP) array for tumor and matched normal samples were imported into dChip [31]. Copy number profiles were calculated by (a) normalizing the intensities using an invariant set of probes, (b) calculating a raw copy number for each SNP using “model based expression” with perfect match/mismatch difference, and (c) using median smoothing with a 10 SNP window. To eliminate batch specific noise in the copy number data, the calculations were performed in smaller batches based on the creation date of the CEL-files. The copy number data was segmented and analyzed within each cluster defined by the extent of PDGFRA or EGFR expression using GLAD [32] and GISTIC2.0 [33] at an amplitude threshold of ±0.2 as implemented in the web application of Gene Pattern at http://genepattern.broadinstitute.org.

### Survival analysis

Overall survival time was separately calculated for patients grouped according to the clusters of PDGFRA expression in glioma samples from the date of surgery until death or the last follow-up contact. The clinical data for the Rembrandt data set was downloaded in May 2012. Kaplan-Meier survival curves were generated and analyzed with log-rank test in SPSS software.

### Isolation and culture of glioma cells

Glioma biopsies of fresh tumor tissue were obtained from patients operated at the Clinic of Neurosurgery, Lund University Hospital, Sweden. Written informed consent was obtained from patients. For preparing viable glioma cells, the fresh specimen were cut into small pieces, and incubated in IMDM with 0.5 mg/ml collagenase (Sigma) and 25 mg/ml DNAse (Sigma) at 37°C for 40 minutes. Red cells were lysed with NH_4_Cl. The remaining glioma cells were washed in PBS containing 2% fetal calf serum (FCS). Glioma cells were grown in poly-L-Lysine (PLL) coated flasks with DMEM/F12 (1∶1) medium (Life Technologies) supplemented with 2% FCS, D-(+)-Glucose solution (0.6%; Sigma), heparin (5 µg/ml; Sigma), sodium bicarbonate (0.1%; Sigma), N2 supplement (Life Technologies), FGF2 (20 ng/ml; Peprotech), sonic hedgehog (SHH) (2 ng/ml of C24II version, R&D Systems), and PDGF-AA(20 ng/ml; Peprotech).

### Flow cytometric analysis

About 10^5^ to 10^6^ cells were stained with allophycocyanin (APC)-conjugated anti-CD44 monoclonal antibody (mAb, clone C26, BD) in combination with phycoerythrin (PE)-conjugated mAb against PDGFRA (clone αR1, BD) for 10 minutes in the dark at 4°C. Subsequently, cells were washed once with PBS and suspended in 500 µl PBS supplemented with 2% FCS and 1.0 mg/ml 7- aminoactinomycin D (7-AAD, Sigma). PDGFRA and CD44 expression was evaluated using a FACSCalibur (Becton Dickinson Immunocytometry Systems) and analyzed using FlowJo software.

### Fluorescence *in situ* hybridization (FISH) analysis of *PDGFRA* copy-numbers

FISH analysis of *PDGFRA* copy-number in metaphase and interphase cells was performed in glioma cells cultured in the medium described above according to standard protocols [34], using a fluorescein-labelled probe for PDGFRA together with a proximal (CHIC2) Cy3-labelled control probe for chromosome 4 (Kreatech Biotechnology/Medprobe, Lund, Sweden). Gene amplification was defined as the presence of at least double the number of *PDGFRA* probe signal compared to the control probe. Ploidy was defined by the number of signals of the control probe (2 signals = 2n, 4 signals = 4n, etc.).

### Bromodeoxyuridine (BrdU)-based cell proliferation assay

L2 glioma cells, a cell line previously tested capable of generating xenograft tumors (the Lux-2 cells in [35]), were seeded (2×10^5^ cells/well) into 6-well plates and cultured with (+) or without (−) FGF2 (20 ng/ml) for 48 h. Cells were then incubated with 10 µM BrdU for 1 h. Proliferating cells with BrdU incorporated were estimated using BD Pharmingen™ BrdU Flow Kits according to the manufacture's protocol (BD).

### RT-PCR analysis

Total mRNA from the glioma cells was prepared using RNAeasy kit (Qiagen) in accordance with the manufacturer's protocol. Twenty microliters of cDNA was synthesized using 1 µg total mRNA within SuperScript First-Strand Synthesis System (Life Technologies) according to the manufacturer's instructions. Two microliters of cDNA were amplified by PCR in a 50-µl-reaction mixture volume containing 2.5 mM deoxynucleoside triphosphate mix, 10 mM specific primers, and 2.5 U Taq DNA polymerase (Life Technologies). After an initial denaturation at 94°C for 3 minutes, 25 cycles were performed at 94°C for 30 s, 57°C for 40 s, and 72°C for 30 s. As a control, glyceraldehyde-3-phosphate dehydrogenase (GAPDH) mRNA levels were estimated by RT-PCR at 25 cycles. The primers used in this study were: PDGFRA, 5′-CTAATCCTCTGCCAGCTTTC-3′ and 5′-TCACTTCCAAGACCGTCAC-3′; GAPDH, 5′- GTCGGAGTCAACGGATT-3′and 5′- AAGCTTCCCGTTCTCAG-3′.

### Immunohistochemical staining of PDGFRA and FGF2 expression

Formaldehyde-fixed, paraffin-embedded tissue blocks derived from surgical excision biopsies were obtained from the archive of Department of Pathology, Lund University Hospital. Permission for using the material was obtained from The Regional Ethical Review Board in Lund. Sections of five-µm thickness were mounted on capillary glass slides (DAKO ChemMate Capillary Gap Microscope Slides, 75 mm, DAKO Sweden AB). All sections were microwave pre-treated in 10 mM citrate buffer pH 6.0 for 15 minutes at 800 W in order to achieve antigen retrieval. An automated immunostainer (TechMateTM 500 Plus, DAKO Sweden AB) was used for the staining procedure using DAKO ChemMate Kit Peroxidase/3-3′ diaminobenzidine. Primary antibodies used were polyclonal for anti-FGF2 (Sigma-Aldrich, 1∶ 2000 dilution) and clone D1E1E for anti-PDGFRA (Cell Signaling, 1∶400 dilution). We analyzed a collection of samples containing 6 low-grade gliomas (two pilocytic astrocytomas, three astrocytomas grade II and one oligoastrocytoma grade II) and 15 high-grade gliomas (one anaplastic astrocytoma grade III, one anaplastic oligodendroglioma grade III, one anaplastic oligoastrocytoma grade III and 12 GBM). The staining was semi-quantitatively evaluated for staining intensity within a defined area. The staining was judged as markedly positive staining (++), low-moderately positive staining (+) or no positive staining (−). In each section, the predominant staining intensity was noted in the grading score.

## Results

**Figure 1:**
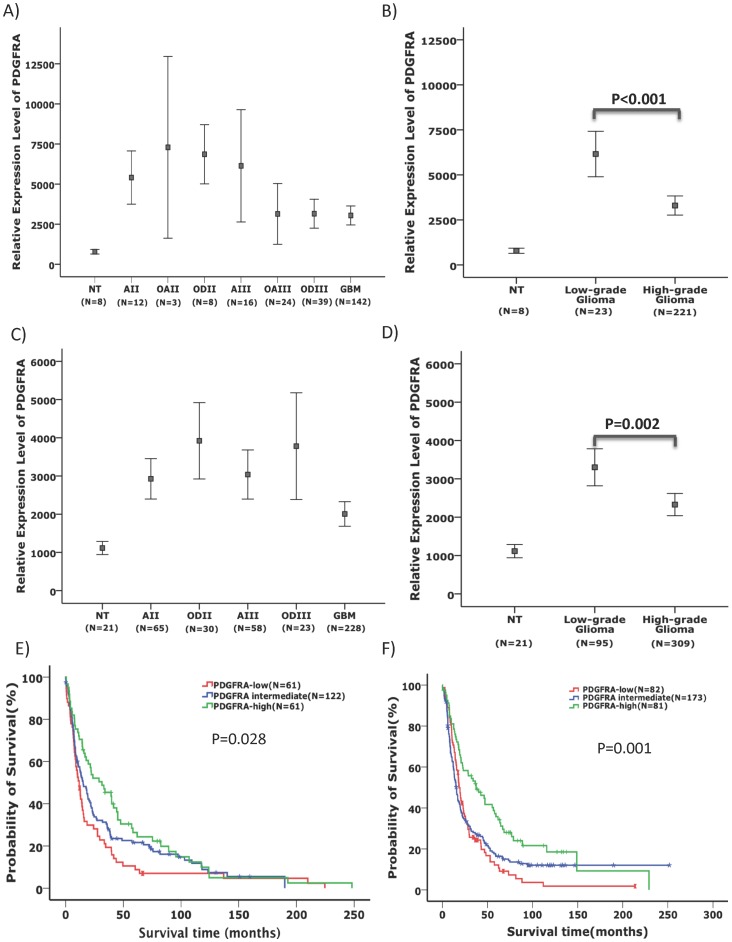
Differential expression of PDGFRA in glioma subtypes and its association with patient survival time. In both GSE16011 (**A, B**) and Rembrandt (**C, D**) data sets, PDGFRA expression (Mean ± 2 SEM, SEM: standard error of the mean) was highly variable among the samples of each morphological subtype (**A** and **C.** NT: non-tumor; AII, astrocytoma grade II; AIII: astrocytoma grade III; GBM, glioblastoma; OD: oligodendroglioma; OA: oligoastrocytoma). Low-grade gliomas showed significantly higher levels of PDGFRA expression compared to high-grade gliomas (**B** and **D**). Kaplan-Meier plots showed that in both GSE16011 (**E**) and Rembrandt (**F**) data sets, the survival of patients with the upper 25% PDGFRA expression (PDGFRA-high) in glioma samples irrespective of their morphological diagnosis was significantly longer compared with those glioma patients with PDGFRA expression at the lowest 25% (PDGFRA-low) and the intermediate 50% (log-rank test).

To characterize the differential PDGFRA expression patterns in the various glioma subtypes, we compared PDGFRA expression among glioma morphological subtypes in GSE16011 and Rembrandt data sets including 244 and 404 gliomas of known diagnosis, respectively. High range of variations in PDGFRA expression was evident in each morphological subtype. In both data sets, the expression of PDGFRA was enriched in low-grade (grade II) gliomas compared to high-grade (grades III and IV) gliomas (p<0.001 and p = 0.002 in GSE16011 and Rembrandt, respectively, *t* test, **Figure 1A, 1B, 1C, 1D**). Morphological diagnosis of gliomas is known to be subjective and inconsistent between the observers [36], which may account for the large ranges of PDGFRA expression among the samples within each morphological subtype, we thus analyzed PDGFRA expression in glioma samples independent of their morphological diagnosis. We analyzed the survival and clinical diagnoses in gliomas with the upper 25% PDGFRA expression levels (PDGFRA-high gliomas) and compared to gliomas with lower 25% levels of PDGFRA expression (PDGFRA-low gliomas). Both the PDGFRA-high and PDGFRA-low groups contained gliomas of all morphological subtypes (**Table 1**). The frequency of *IDH1* mutation was significantly higher in PDGFRA-high gliomas compared to PDGFRA-low gliomas.

**Table 1:** Pathological and clinical parameters of gliomas with varying extent of PDGFRA expression.

Data set	Morphological Subtypes	Number of samples		
		PDGFRA-low (N = 61)	PDGFRA intermediate (N = 122)	PDGFRA-high (N = 61)
GSE16011 (N = 244)	AII	0	7	5
	AIII	0	8	8
	OAII	0	1	2
	OAIII	10	10	4
	ODII	0	1	7
	ODIII	7	23	9
	GBM	44	72	26
	IDH1 mutation	14/52 (P = 0.02)*	35/94	24/48
	1p LOH	9/31 (P = 0.001)*	25/69 (P<0.001)*	18/34
	19q LOH	9/30 (P = 0.005)*	22/70 (P<0.001)*	16/30
	EGFR amplification	13/35 (P<0.001)*	19/65 (P<0.001)*	5/32
	Age at diagnosis (Median ± SD)	55.2±13.17 (P<0.001)*	51.51±13.95 (P = 0.005)*	43.89±13.68
	Survival years (Median ± SD)	0.98±3.53 (P = 0.045)*	1.27±3.26 (P = 0.13)*	2.41±3.88
Rembrandt (N = 336)**	Morphological Subtypes	PDGFRA-low (N = 82)	PDGFRA intermediate (N = 173)	PDGFRA-high (N = 81)
	AII	4	29	17
	AIII	13	18	16
	ODII	3	10	9
	ODIII	4	14	6
	GBM	58	102	33
	1p LOH	15/51 (P<0.001)*	27/103 (P<0.001)*	26/51
	19q LOH	8/51 (P<0.001)*	25/103 (P<0.001)*	23/51
	EGFR amplification	32/51 (P<0.001)*	59/103 (P<0.001)*	10/51
	Survival years (Median ± SD)	1.53±2.46 (P = 0.002)*	1.23±3.21 (P = 0.007)*	3.04±3.49

PDGFRA expression among the glioma samples in GSE16011 and the Rembrandt data sets was analyzed independent of morphological diagnosis. The samples with the upmost 25% of PDGFRA expression (PDGFRA-high) were compared with the samples with the lowest 25% of PDGFRA expression (PDGFRA-low) and the samples with the 50% intermediate levels of PDGFRA expression (PDGFRA intermediate) regarding frequencies of IDH1 mutation, age at diagnosis and survival period. The data on IDH1 mutation, LOH at 1p and 19q, and EGFR amplification for GSE16011 data set were derived from the results of Gravendeel et al [27]. The data on LOH in regions of 1p36 (1p36.23 or 1p36.32 or 1p36.31) and 19q13 (19q13.32 or 19q13.41) as analyzed by Mariani *et al.* [47], and EGFR amplification for the Rembrandt data set were derived from GISTIC2.0 analysis of the 50K *HindIII* SNP array data [28]. The differences for *IDH1* mutation rate, LOH at 1p and 19q and EGFR amplification were analyzed using chi-square test. The median age at diagnosis and survival period between the PDGFRA subgroups were analyzed using Fisher's exact test, One-way Anova and log-rank tests, respectively.

* : Comparison with the PDGFRA-high group.

** : The number of patients with known survival data.

Furthermore, compared to PDGFRA-low and PDGFRA-intermediate gliomas, PDGFRA-high gliomas were associated with significantly higher frequency of loss of heterozygosity (LOH) at 1p and 19q. Conversely, PDGFRA-low and PDGFRA-intermediate gliomas were associated with significantly higher frequency of EGFR amplification compared to PDGFRA-high gliomas (**Table 1**). Interestingly, in both the GSE16011 and the Rembrandt data sets, PDGFRA-high gliomas were associated with younger age at disease onset compared to PDGFRA-low gliomas (p<0.01, *t* test, **Table 1**). Patients with PDGFRA-high gliomas survived significantly longer compared to patients with PDGFRA-low gliomas (p = 0.028 and p = 0.001 in GSE16011 and Rembrandt, respectively, log-rank test, **Figure 1E, 1F** and **Table 1**). We further assessed if this is true for patients with gliomas within the same malignancy grade. In the Rembrandt data set, patients with PDGFRA-high GBMs or PDGFRA-high low-grade gliomas showed significant better survival and younger age at disease onset compared to patients with PDGFRA-low GBMs or PDGFRA-low low-grade gliomas, respectively (**Figure S2**).

**Figure 2:**
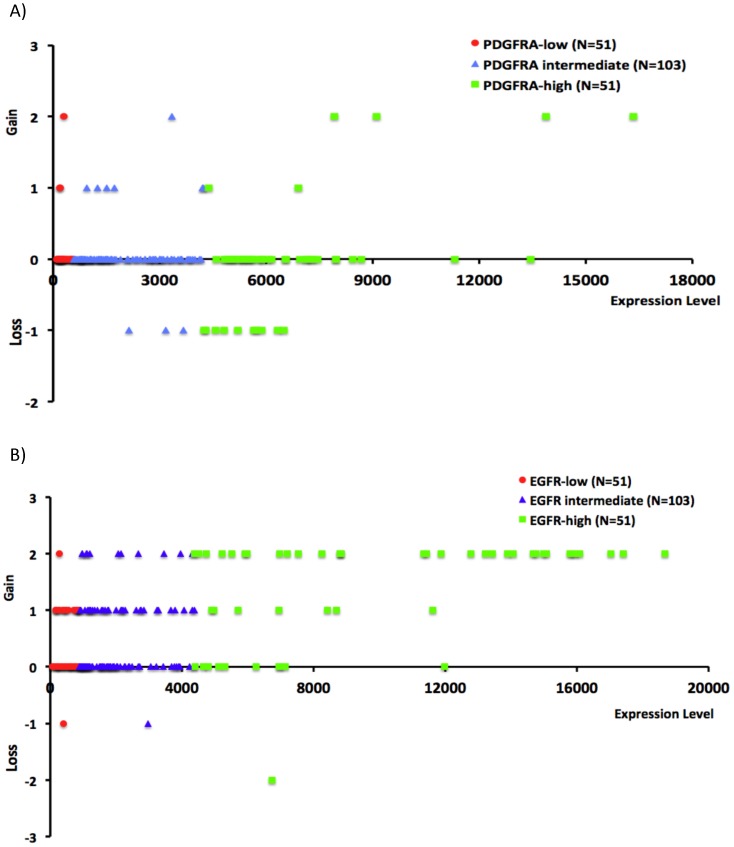
Infrequent association between enriched PDGFRA expression and *PDGFRA* amplification in glioma genomes. The SNP data in the Rembrandt data set were analyzed for the gain or loss of *PDGFRA* (**A**) and *EGFR* (**B**) using dChip and GISTIC2.0 with glioma samples grouped according to the relative levels of PDGFRA or EGFR expression. In each plot, the X-axis indicates the relative levels of PDGFRA or EGFR expression, and the Y-axis indicates the extent of *PDGFRA* or *EGFR* gain or loss (2, 1, 0, −1 and −2 for above 3.7 copies of genes, 2.3–3.7 copies of gene, no detectable alteration, heterozygous loss, and homozygous loss, respectively).

To address the causes of PDGFRA overexpression in gliomas, we first analyzed the 50k *HindIII* SNP data in the Rembrandt data set using dChip and GISTIC2.0. Six of the 51 gliomas in the PDGFRA-high group showed gain of *PDGFRA*, and heterozygous loss of *PDGFRA* gene was found in 10 cases in this group (**Figure 2A**). This was in striking contrast to the nearly invariable association between gain of *EGFR* and enriched EGFR expression in the same cohort of glioma samples (**Figure 2B**). PDGFRA overexpression is thus unlikely caused by *PDGFRA* gene amplification in the majority of gliomas.

**Figure 3:**
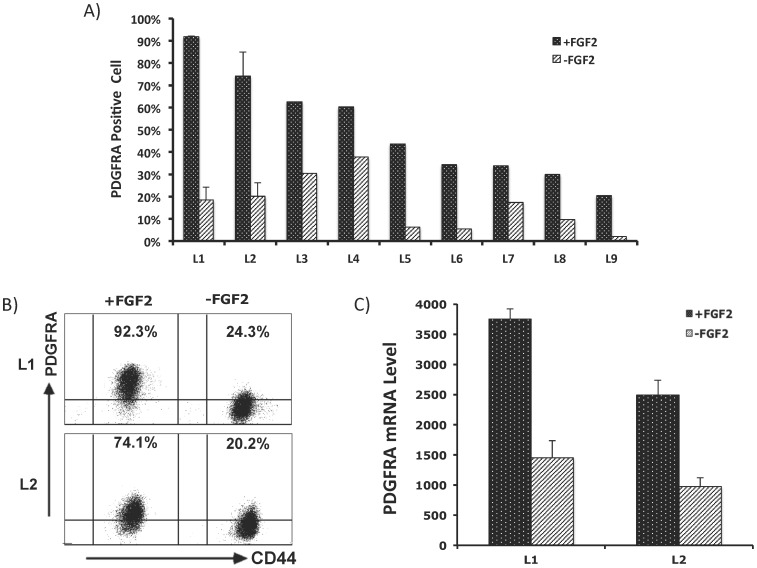
FGF2-dependent maintenance of PDGFRA expression. **A**) Percentages of PDGFRA-positive cells in the indicated glioma cell lines (L1–L9) following a culture for 7 to 10 days with (+) or without (−) FGF2 support. **B**) Maintenance of PDGFRA expression in glioma cells (L1 and L2) cultured with the support of FGF2 alone for 7 days. In contrast to PDGFRA, the expression of CD44 was not affected by FGF2. **C**) RT-PCR detection of PDGFRA expression in the parallel cultures as in **B**).

**Figure 4:**
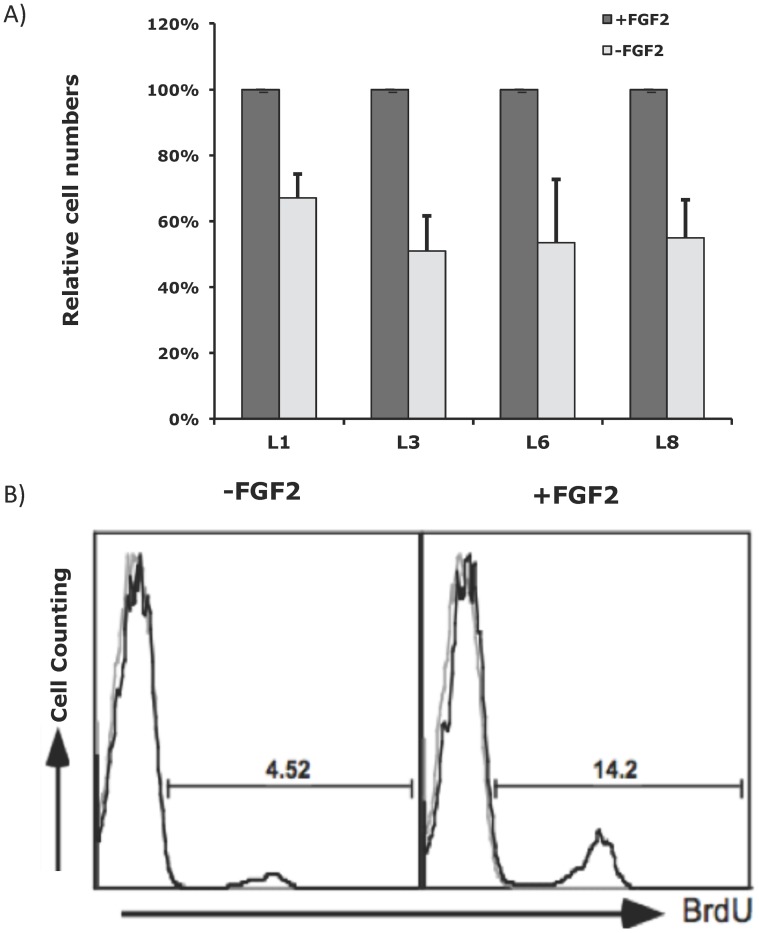
FGF2-dependent cell proliferation *in vitro*. **A**) Low-passage glioma cell lines were cultured in a growth factor cocktail consisting of SHH, PDGFA and FGF2 (+FGF2), or only SHH and PDGFA (−FGF2). The relative cell numbers after one passage of culture for 7 to 10 days are shown under the 2 conditions. **B**) L2 cells were cultured for 7 days with (+) or without (−) FGF2 support alone. The percentages of S-phase cells were evaluated by BrdU assays.

Next, we hypothesized that PDGFRA overexpression in glioma cells is niche factor dependent. We established 11 low passage glioma cell lines in N2 supported DMEM/F12 medium containing 2% FCS with a growth factor cocktail consisting of FGF2, PDGF-AA and SHH. PDGFRA expression was measured in fresh cells and also monitored in cultured cells using flow cytometry. With a cut-off at >4 copies, FISH analysis did not detect *PDGFRA* amplification in any of these cell lines (**Table 2**). Under our culture conditions, PDGFRA expression was maintained in all cell lines as detected in flow cytometric analysis. In all of the 9 cell lines tested, withdrawal of FGF2 resulted in a drop of PDGFRA expression (**Figure 3**). However, the expression of CD44, a marker of glial progenitor cells widely expressed on the surface of glioma cells [37], was not detectably affected. Thus, withdrawal of FGF2 did not result in a general decline of gene expression in glioma cells. No measurable decline in PDGFRA expression was detected following withdrawal of PDGFA or SHH (data not shown). A decline in PDGFRA expression following withdrawal of FGF2 was concordant with diminished cell proliferation, as assessed by cell counting and BrdU incorporation assays (**Figure 4**). Thus, FGF2 appears to be a key growth factor involved in regulating PDGFRA expression in glioma cells.

**Table 2:** No association between PDGFRA expression and *PDGFRA* amplification in glioma cells as detected by FISH assay.

Patient ID (Age/Gender)	Pathological Diagnosis	*PDGFRA* copy number	Ploidy	Karyotype	PDGFRA expression
L1 (66/M)	GBM	2	2n	*	UD
L3 (49/M)	GBM	2	2n	**	+
L4 (60/M)	Astrocytoma II	2	2n	45,X, -Y [24]/46,XY [1]	UD
L5 (45/F)	Oligoastrocytoma II	2	2n	44–45,X, -X [cp3]/46,XX [22]	+
L6 (36/M)	Astrocytoma II	2	2n	45,X, -Y [13]/46,XY [10]	+
L8 (7/F)	Pilocytic astrocytoma	2	2n	46, XX	+
L10 (31/M)	Oligodendroglioma III	2	2n	46, XY	UD
L11 (8/M)	Astrocytoma III	2	2n	46, XY	+
L12 (36/M)	GBM	2	2n	45, X, -Y [11]/46, X, -Y, +7 [3]/46, XY [10]	+
L13 (58/F)	GBM	4	4n	Not done	+
L14 (65/M)	GBM	2	2n	45,X, -Y [14]/46,X, -Y, +7 [3]/46,XY [6]	+

Freshly isolated glioma cells were stained with anti-CD45 and anti-PDGFRA mAbs and analyzed by flow cytometry. The remaining cells were cultured under +FGF2 condition, karyotyped and also assessed for *PDGFRA* copy numbers with FISH analysis. The FGF2 dependent PDGFRA expression was observed in cells derived from patients L3, L5, L6, L8, L11, L12, L13, and also L14. Cells from L1, L4 and L10, which initially did not express PDGFRA, expressed PDGFRA following FGF2 supported culture protocol.

* : 41–42,X, -Y, der (1) t (1; 17)(p36; q21), add (4)(q31), del (6)(q21), +7, −10, −11, −13, −14,? dup (14)(q12q22),−15,−17,+2mar,inc[cp9]/80–84,idemx2[cp16].

** : 46,XY, ins (1; 4)(p36; q31q25), t (1; 12)(q21; p11) [18]/46,XY, add (1)(p36), del (10)(q24), del (15)(q24), −17, +mar [7]. UD: undetectable.

**Figure 5:**
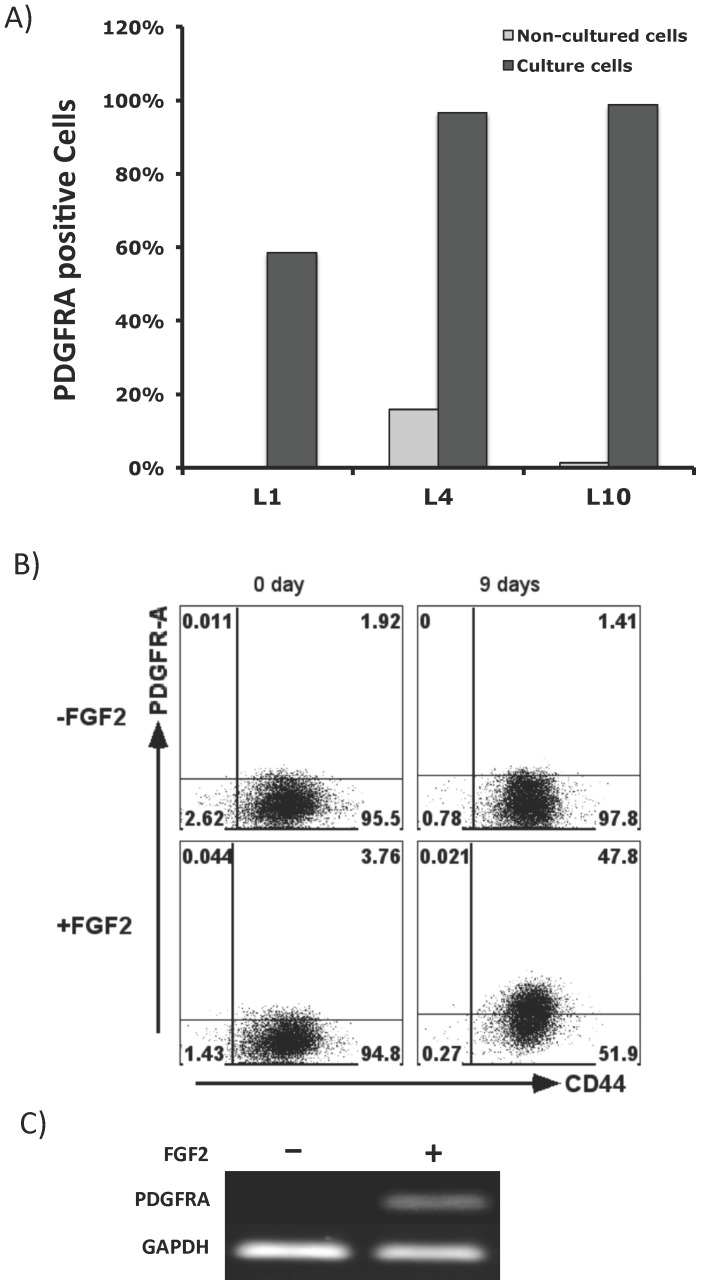
Induction of PDGFRA expression in glioma cells. **A**) Fresh cells from 3 different glioma samples without detectable PDGFRA expression cultured with the support of FGF2 for 2 to 3 weeks were analyzed for PDGFRA expression and compared with the fresh cells from the same patients simultaneously. **B**) and **C**) L2 cells from xenograft gliomas [35] were cultured for 9 days under +FGF2 or −FGF2 conditions and assessed for PDGFRA expression by flow cytometry and RT-PCR analysis, respectively. Non-cultured cells served as control (0 days).

We further assessed whether FGF2 can induce PDGFRA expression in glioma cells. Fresh cells from 3 specimens with undetectable PDGFRA expression in flow cytometric analysis were selected (**Table 2** **)**. Following a culture period of between 2 and 3 weeks, PDGFRA expression was re-analyzed when sufficient cells were available. Compared to the uncultured cells analyzed simultaneously, cultured cells from all 3 glioma specimens showed enhanced levels of PDGFRA expression (**Figure 5A**). And, PDGFRA expression was maintained when these cultures were passaged. This enhanced PDGFRA expression was unlikely due to a selection of normal glial progenitor cells, because karyotype abnormalities were also detected in cells derived from the parallel cultures (**Table 2**). Further, PDGFRA expression was also induced by FGF2 in a xenograft GBM cell line [35], as detected at the protein and mRNA levels by flow cytometry and RT-PCR assays (**Figure 5B, 5C**).

**Figure 6:**
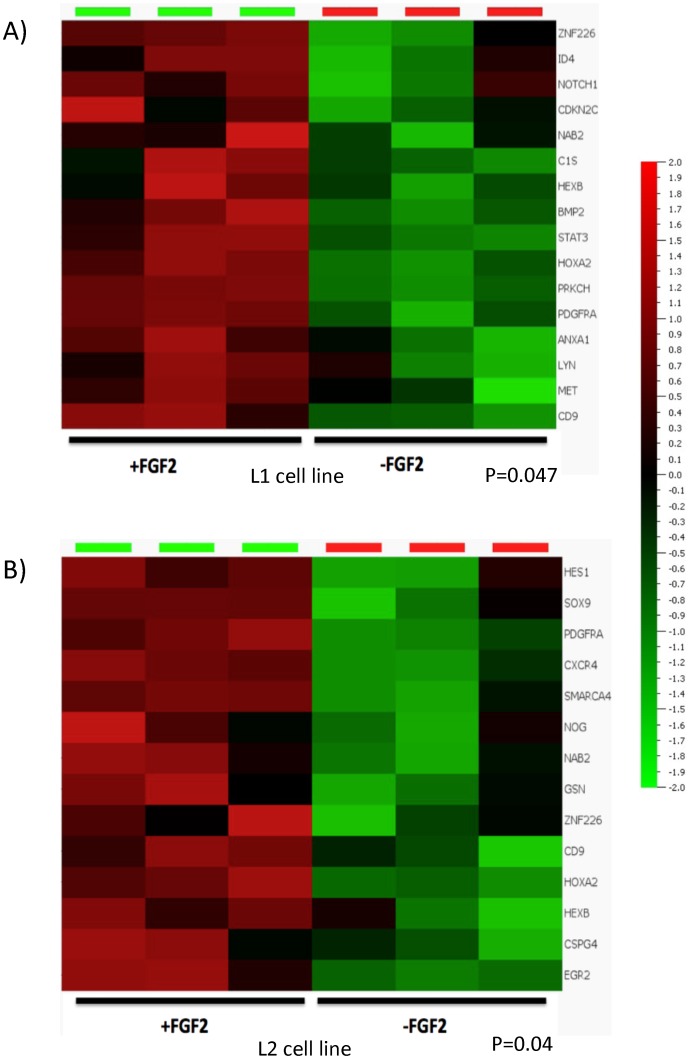
FGF2 maintained the expression of a subset of gliogenic genes in glioma cells. Two glioma cell lines (L1 and L2) were cultured under +FGF2 or −FGF2 conditions. PDGFRA expression was undetectable in −FGF2 samples as assessed by flow cytometry; mRNA from three replicate samples under each of the two conditions were extracted and prepared for analysis with GeneChip human gene ST array. Data shown are the differential expression of subset of gliogenic genes under +FGF2 or −FGF2 conditions.

To study whether FGF2 affects the expression of other gliogenic genes in addition to PDGFRA, we characterized the transcriptome profiles in two glioma cell lines cultured under the same conditions as above with or without FGF2 support. A glioma-relevant gliogenesis gene list was generated (**Figure S1**) and its expression signature was assessed in the transcriptome data of the 2 cell lines. In addition to PDGFRA, a subset of gliogenic genes including CD9, HEXB, HOXA2, NAB2, and ZNF226 were enriched at significant levels in FGF2 supported culture conditions (**Figure 6**). These genes are known to be involved in the development of myelinogenic progenitor cells [38], or are reported as responders of FGF signaling [39].

**Figure 7:**
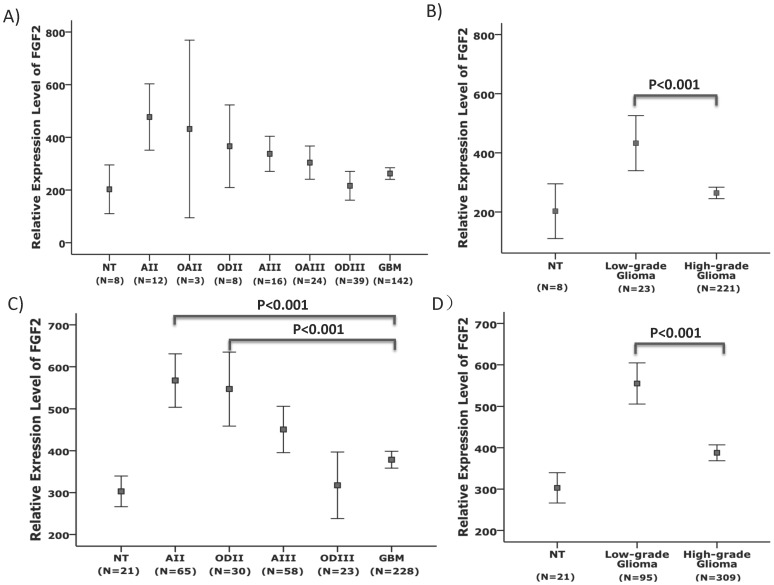
Enriched FGF2 expression in low-grade gliomas. The relative expression levels of FGF2 (Mean ± 2SEM) were analyzed in the GSE16011 (**A** and **B**) and the Rembrandt (**C** and **D**) data sets according to the morphological diagnosis of gliomas (**A** and **C**). Low-grade gliomas showed significantly higher levels of FGF2 expression compared to high-grade gliomas (**B** and **D**, *t* test).

To address whether the expression patterns of FGF2 and PDGFRA are correlated *in vivo*, we analyzed FGF2 expression in GSE16011 and the Rembrandt data sets. In both data sets, the patterns of FGF2 expression were similar to those of PDGFRA expression; FGF2 expression was significantly enriched in low-grade gliomas, compared to high-grade gliomas (P<0.001, *t* test, **Figure 7**).

**Figure 8:**
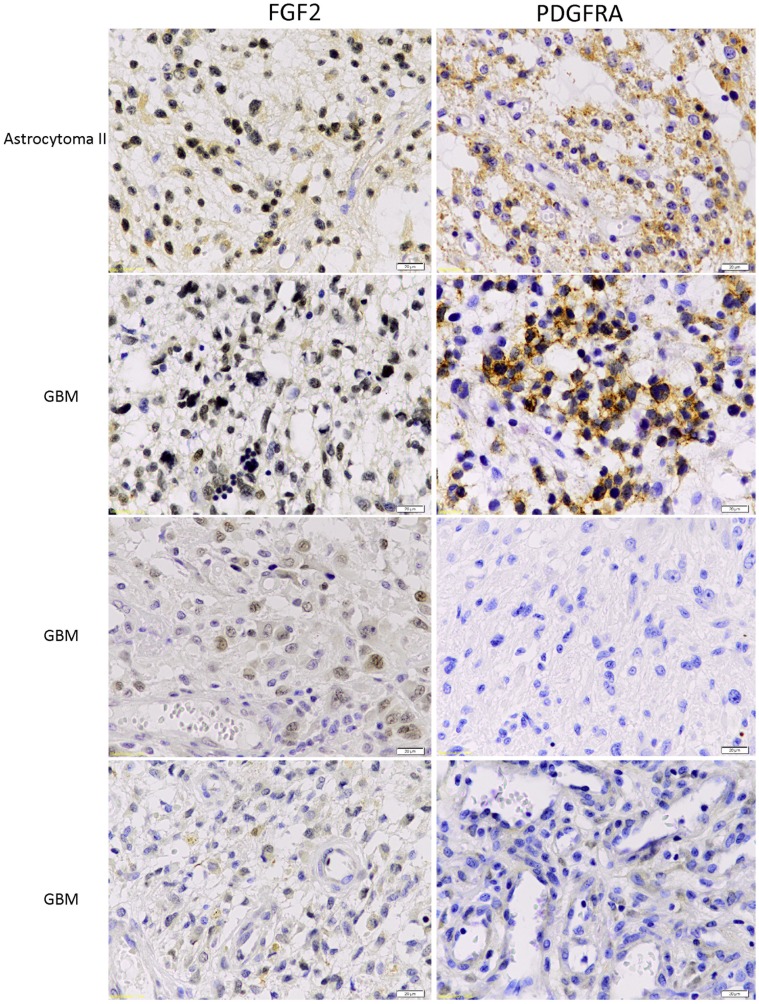
Concordant patterns of FGF2 and PDGFRA immunohistochemical staining in glioma samples. Consecutive sections for each glioma samples were used for the staining of FGF2 and PDGFRA. Staining results of representative glioma samples are shown. In all samples, a correlation between the extent of FGF2 and PDGFRA staining was observed. The scale bars denote 20 µm.

Further, we performed immunohistochemical staining of FGF2 and PDGFRA expression in cohort of 6 low-grade and 17 high-grade glioma samples (**Table 3**). In two GBM samples, immunostaining of both FGF2 and PDGFRA was not detectable. For both FGF2 and PDGFRA (**Figure 8**), region-dependent focal staining was seen in the remaining samples. In general, FGF2 staining appeared to be more diffuse compared to the staining of PDGFRA. Both intracellular and cell membrane staining of PDGFRA was observed while FGF2 exhibited a nuclear and/or a cytoplasmic staining. The staining intensity varied between cases, but a correlation of FGF2 and PDGFRA was observed. Cases with weak staining intensity of FGF2 showed weak or no staining of PDGFRA.

**Table 3:** Immunohistochemical staining results for PDGFRA and FGF2 in glioma samples - Predominant staining intensity in each sample.

Patient ID	Pathological Diagnosis	PDGFRA	bFGF
1	Oligoastrocytoma II	**+**	**+**
2	Pilocytic astrocytoma	**+**	**+**
3	Astrocytoma II	**+**	**+**
4	Astrocytoma II	**+**	**+**
5	Astrocytoma II	**+**	**++**
6	Pilocytic astrocytoma	**+**	**+**
7	GBM	**−**	**+**
8	GBM	**+**	**+**
9	Oligodendroglioma III	**+**	**+**
10	GBM	**−**	**−**
11	GBM	**++**	**+**
12	GBM	**++**	**+**
13	Astrocytoma III	**+**	**+**
14	GBM	**+**	**+**
15	GBM	**+**	**+**
16	GBM	**+**	**+**
17	GBM	**+**	**+**
18	GBM	**+**	**+**
19	GBM	**+**	**+**
20	GBM	**+**	**+**
21	Oligoastrocytoma III	**+**	**+**
22	GBM	**++**	**+**

**++:** Markedly positive staining. **+:** Low-moderately positive staining. **−:** No positive staining.

## Discussion

Receptor tyrosine kinases that play crucial roles in glial genesis and glioma development have been suggested as potential therapeutic targets against gliomas [5]. However, extensive clinical studies have failed to demonstrate the efficacy of kinase inhibitors in GBM patients [40]. A better understanding on the correlation and the causes of receptor tyrosine kinase overexpression in glioma subtypes may improve the utility of kinase inhibitors in combating gliomas.

The occurrence of PDGFRA expression and its association with the pathological and clinical parameters of gliomas have been controversial [8], [14], [24], [25], [26]. Using two large data sets consisting of 648 glioma samples of known morphological diagnosis, we found that under the supervision of morphological diagnosis, PDGFRA expression was enriched in low-grade gliomas compared to high-grade gliomas. However, all morphological subtypes were represented among gliomas with enriched PDGFRA expression. These gliomas were significantly associated with frequent *IDH1* mutation, younger age at disease onset and better survival outcome compared to the gliomas with lower levels of PDGFRA expression. This may be due to the inconsistencies in morphological diagnosis [36]. Alternatively, gliomas overexpressing PDGFRA may represent a separate molecular entity that is independent of morphological diagnosis.

Our findings extend previous reports [26], [41], [42] that irrespective of morphological diagnosis, gliomas with high level PDGFRA expression are associated with concomitant *IDH1* mutation, higher frequency of deletions at 1p and 19q, lower frequency of EGFR amplification, younger age at diagnosis and better patient's survival. However, different mechanisms may account for high-level PDGFRA expression in gliomas. EGFR overexpression in gliomas is invariably associated with *EGFR* gene amplification [23]. *PDGFRA* gene amplification and mutation in gliomas with high expression levels was also reported previously [43], [44]. Our findings showed that in adulthood gliomas as analyzed in this report, amplification of *PDGFRA* gene was unlikely the main cause of PDGFRA overexpression in gliomas. Instead, our findings in cell culture studies and expression analysis in glioma samples supported the hypothesis that PDGFRA expression was dependent on the niche factors in gliomas. However, correlated expression between FGF2 and PDGFRA at the mRNA level was not observed in both the Rembrandt and GSE16011 data sets (data not shown). Thus, FGF2-dependent PDGFRA expression as observed in our study is likely an indirect effect; FGF2-dependent PDGFRA expression is probably applicable only to a specific subset of gliomas. Our analyses on hallmark alterations in glioma genome show that LOH of 1p and 19q is more frequent in PDGFRA-high gliomas, and that EGFR amplification is more frequent in PDGFRA-low and PDGFRA-intermediate gliomas. Previous studies demonstrate that gliomas with deletion at both 1p and 19q and gliomas with EGFR amplifications are mutually exclusive regarding their cell(s) of origin, profiles of transcriptomic and genomic alterations, and clinical characteristics [45], [46], [47], [48], [49], [50]. Thus, although further in-depth characterizations on their transcriptomic and genomic profiles are required, gliomas with varying extent of PDGFRA expression could represent different molecular subtypes that respond differently to FGF2 signaling.

FGF2 is widely expressed in normal brain astrocytes and also in gliomas [8], [51], [52], [53], [54]. In large numbers of glioma samples, our results showed that the pattern of FGF2 expression was similar to that of PDGFRA expression. In the low-passage cell lines tested in our study, FGF2 maintained PDGFRA expression *in vitro*. Further, FGF2 was able to induce PDGFRA expression.

Maintenance of PDGFRA expression was concordant with the expression of a subset of gliogenic genes. We speculate that FGF2 mediated signaling can potentially be manipulated to suppress PDGFRA expression and thereby inhibit niche factor-dependent glioma growth.

FGF2-dependent PDGFRA expression appears to be a converged mechanism in normal glial development and glioma genesis. PDGFRA is a characteristic marker of OPC [9], [10]. Early studies had demonstrated an FGF2 dependent PDGF signaling in OPCs [55], [56]. Disruption of PDGFRA signaling results in diminished generation of oligodendrocytes [13]. Enhanced signaling of FGF2 or PDGFRA results in proliferation but blocked differentiation of OPCs towards oligodendrocytes [20], [57], [58]. Although it is beyond the scope of this report, we speculate that additional features governing differentiation and proliferation of oligodendrocyte lineage can be detected in gliomas with enriched PDGFRA expression.

## Supporting Information

Figure S1 **Selection of glioma-relevant gliogenic genes.** A combined glial genesis gene list (172 genes) was created by combining the glial genesis gene list GO:0042063 and the gene list composed of genes controlling oligodendrogenesis [30]. Using unsupervised hierarchical clustering analysis of the expression of glial genesis genes and glioma samples in GSE4290 data set [29]. Seventy-eight gliogenic genes differentially expressed in gliomas were selected after exclusion of genes with standard deviations below 1.5% of the maximal standard deviation in variance filtering. The colored rectangles on the top of the heat map represent morphological diagnosis of glioma samples: orange rectangles for unknown diagnosis; pink for astrocytoma grade II; red for astrocytoma grade III; green for glioblastoma multiforme; blue for oligodendroglioma grade II; yellow for oligodendroglioma grade III and white for non-tumor samples. The glioma-relevant gliogenic genes included: *AGT*, *ANXA1*, *ASCL1*, *ATF5*, *BCL2*, *BMP2*, *C1S*, *CCL2*, *CD86*, *CD9*, *CDK1*, *CDK6*, *CDKN1A*, *CDKN2C*, *CSPG4*, *CTNNB1*, *CXCR4*, *DAG1*, *DLL3*, *EGR1*, *EGR2*, *EIF2B1*, *EIF2B4*, *ERBB2*, *EXOC4*, *FGF2*, *FOXD1*, *GFAP*, *GLI3*, *GPC1*, *GSN*, *HDAC2*, *HES1*, *HES5*, *HEXB*, *HMBS*, *HMGA2*, *HOXA2*, *ID2*, *ID4*, *ITGAM*, *KLF15*, *LAMB2*, *LIF*, *LYN*, *MET*, *MMP14*, *MPP5*, *MYT1*, *NAB2*, *NF1*, *NFIB*, *NOG*, *NOTCH1*, *NR2E1*, *OLIG2*, *PAX2*, *PAX6*, *PDGFRA*, *POU3F2*, *PRKCH*, *PTEN*, *PTPRC*, *RELA*, *SMARCA4*, *SOX11*, *SOX2*, *SOX4*, *SOX5*, *SOX6*, *SOX8*, *SOX9*, *STAT3*, *TCF7L2*, *TGFB2*, *TSPO*, *VCAN*, *ZNF226*.
(TIFF)

Figure S2 **Differential expression of PDGFRA in gliomas of the same malignancy grade is associated with patient survival time.** High-grade (grade III and IV) and low-grade (grade II) gliomas in the Rembrandt data set were separately analyzed for the extent of PDGFRA expression. Kaplan-Meier plots for the survival time of the patients with high-grade gliomas (panel A) or low-grade gliomas (panel B) are shown. Within the same malignancy grade, PDGFRA-high gliomas are associated with better survival; furthermore, the PDGFRA-high gliomas occur more frequently in younger than 40 patients (p<0.01, *X^2^* test, comparing the number of patients younger than 40 and the number of patients older then 60 between the PDGFRA-low and PDGFRA-high group). A similar but not significant trend was observed for the high-grade gliomas in GSE16011 data sets.
(TIFF)
