# Supplementary figures and images for: Better Prognosis of Patients with Glioma Expressing FGF2-Dependent PDGFRA Irrespective of Morphological Diagnosis
Source: PLoS One. 2013 Apr 22;8(4):e61556. doi: 10.1371/journal.pone.0061556 (PMC3632602; doi:10.1371/journal.pone.0061556)

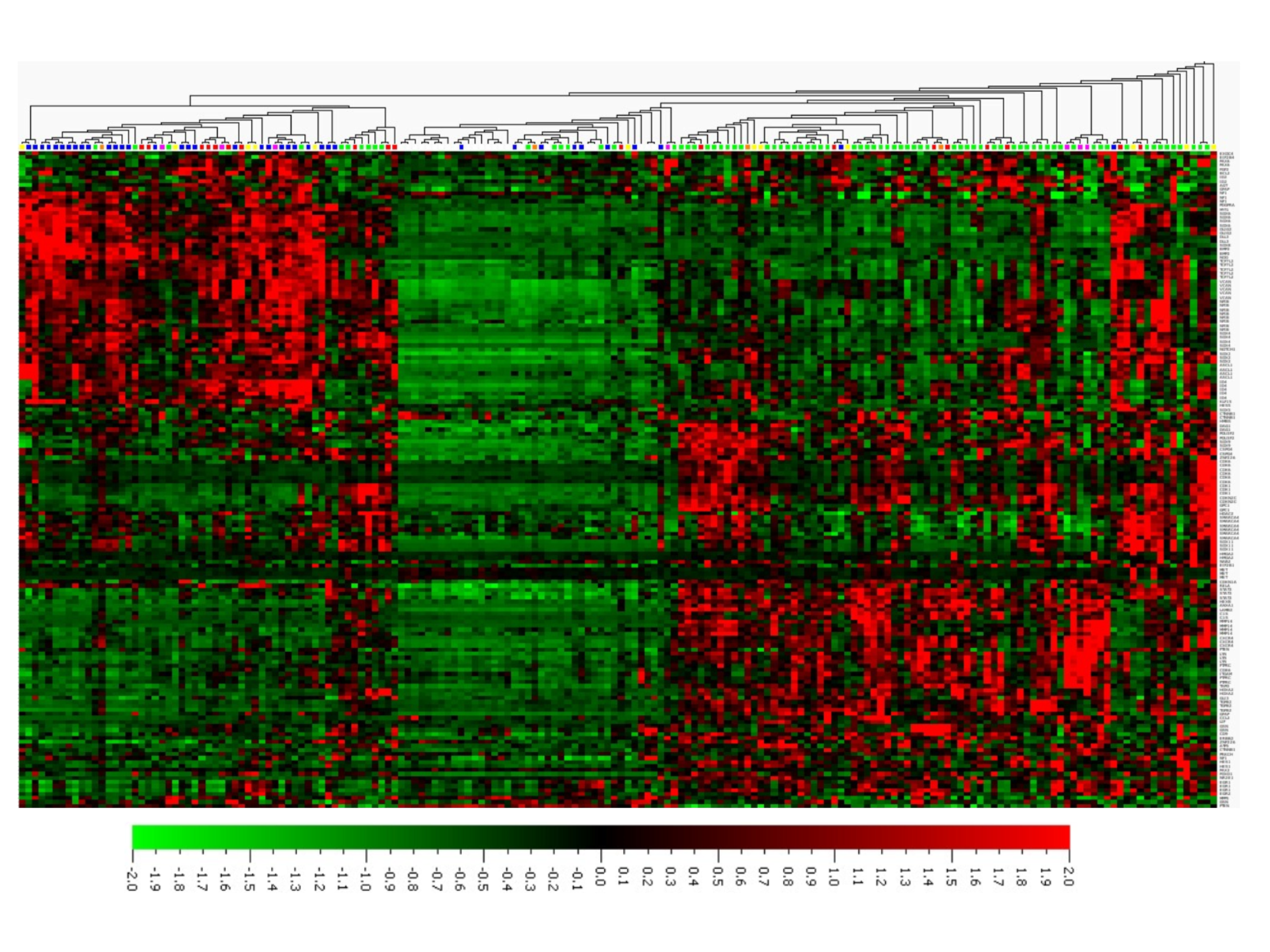

Supplement: Figure S1 — Selection of glioma-relevant gliogenic genes. A combined glial genesis gene list (172 genes) was created by combining the glial genesis gene list GO:0042063 and the gene list composed of genes controlling oligodendrogenesis [30]. Using unsupervised hierarchical clustering analysis of the expression of glial genesis genes and glioma samples in GSE4290 data set [29]. Seventy-eight gliogenic genes differentially expressed in gliomas were selected after exclusion of genes with standard deviations below 1.5% of the maximal standard deviation in variance filtering. The colored rectangles on the top of the heat map represent morphological diagnosis of glioma samples: orange rectangles for unknown diagnosis; pink for astrocytoma grade II; red for astrocytoma grade III; green for glioblastoma multiforme; blue for oligodendroglioma grade II; yellow for oligodendroglioma grade III and white for non-tumor samples. The glioma-relevant gliogenic genes included: AGT, ANXA1, ASCL1, ATF5, BCL2, BMP2, C1S, CCL2, CD86, CD9, CDK1, CDK6, CDKN1A, CDKN2C, CSPG4, CTNNB1, CXCR4, DAG1, DLL3, EGR1, EGR2, EIF2B1, EIF2B4, ERBB2, EXOC4, FGF2, FOXD1, GFAP, GLI3, GPC1, GSN, HDAC2, HES1, HES5, HEXB, HMBS, HMGA2, HOXA2, ID2, ID4, ITGAM, KLF15, LAMB2, LIF, LYN, MET, MMP14, MPP5, MYT1, NAB2, NF1, NFIB, NOG, NOTCH1, NR2E1, OLIG2, PAX2, PAX6, PDGFRA, POU3F2, PRKCH, PTEN, PTPRC, RELA, SMARCA4, SOX11, SOX2, SOX4, SOX5, SOX6, SOX8, SOX9, STAT3, TCF7L2, TGFB2, TSPO, VCAN, ZNF226. (TIFF) [file pone.0061556.s001.tiff]

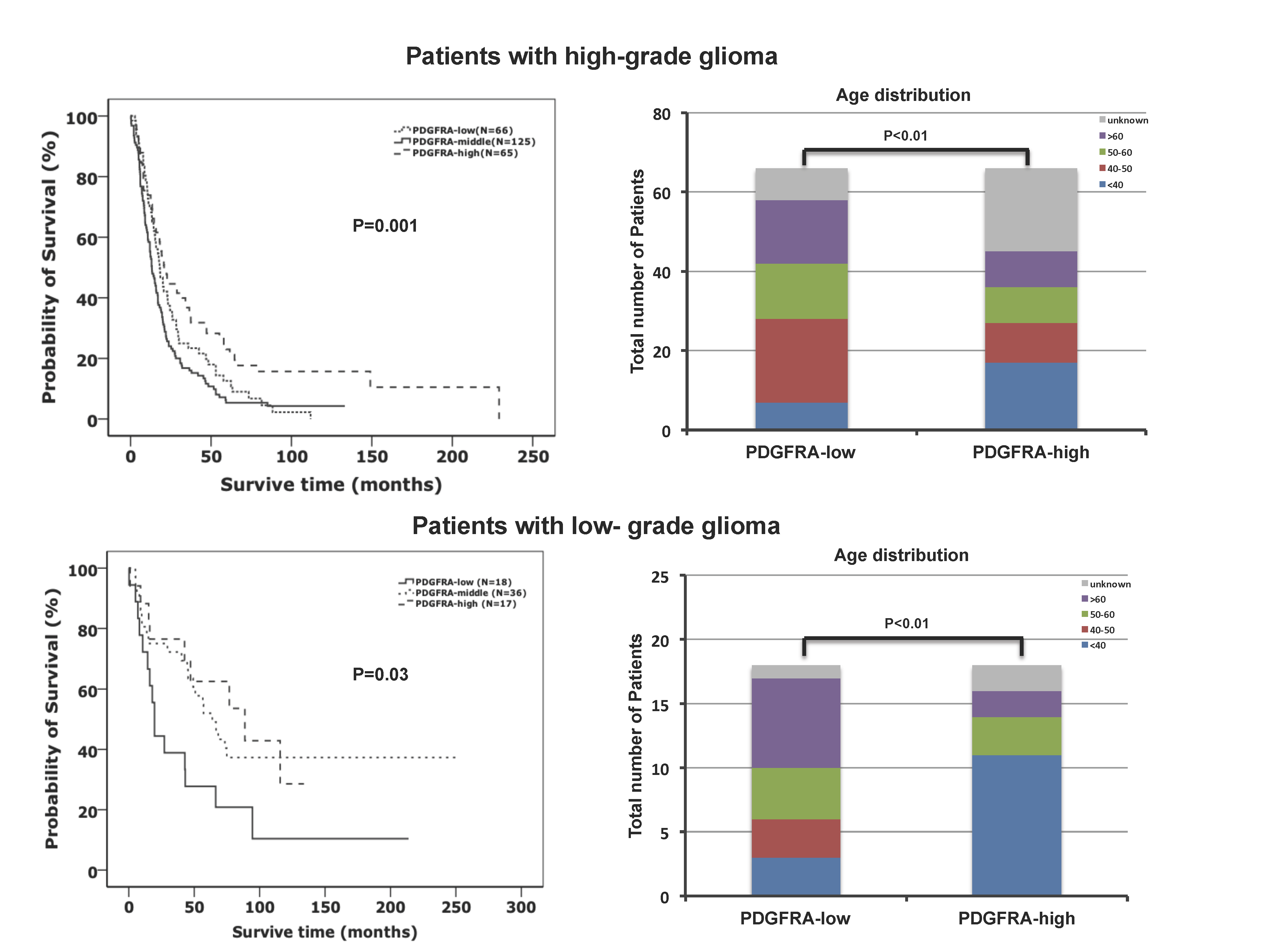

Supplement: Figure S2 — Differential expression of PDGFRA in gliomas of the same malignancy grade is associated with patient survival time. High-grade (grade III and IV) and low-grade (grade II) gliomas in the Rembrandt data set were separately analyzed for the extent of PDGFRA expression. Kaplan-Meier plots for the survival time of the patients with high-grade gliomas (panel A) or low-grade gliomas (panel B) are shown. Within the same malignancy grade, PDGFRA-high gliomas are associated with better survival; furthermore, the PDGFRA-high gliomas occur more frequently in younger than 40 patients (p<0.01, X2 test, comparing the number of patients younger than 40 and the number of patients older then 60 between the PDGFRA-low and PDGFRA-high group). A similar but not significant trend was observed for the high-grade gliomas in GSE16011 data sets. (TIFF) [file pone.0061556.s002.tiff]
